# Metabolic profiling revealed the organ‐specific distribution differences of tannins and flavonols in pecan

**DOI:** 10.1002/fsn3.1797

**Published:** 2020-08-10

**Authors:** Mengyang Xu, Pei Liu, Xiaodong Jia, Min Zhai, Shigang Zhou, Baocheng Wu, Zhongren Guo

**Affiliations:** ^1^ Jiangsu Key Laboratory for the Research and Utilization of Plant Resources Institute of Botany Jiangsu Province and Chinese Academy of Sciences Nanjing China; ^2^ The Jiangsu Provincial Platform for Conservation and Utilization of Agricultural Germplasm Institute of Botany Jiangsu Province and Chinese Academy of Sciences Nanjing China; ^3^ Jiangsu Collaborative Innovation Center of Chinese Medicinal Resources Industrialization Nanjing University of Chinese Medicine Nanjing China

**Keywords:** *Carya illinoinensis*, distribution, flavonols, organ‐specific, tannins

## Abstract

*Carya illinoinensis* is rich in phenolic metabolites such as tannins and flavonols, but both the composition and the distribution of these nutritional constituents in most pecan organs were still unclear. In this experiment, a comprehensive qualification and quantification of phenolic metabolites in eight organs of pecan were conducted for the first time. Ninety‐seven phenolic metabolites were identified, in which twelve were identified for the first time in pecan, including a series of ellagitannins with high molecular weight. Hydrolysable tannin was the dominant kind of phenolic metabolites in pecan. The metabolic profiles of tannins in pecan were extended. Thirty‐three phenolic metabolites were quantified, among them the highest content was ellagic acid pentose in testa. From this experiment, we can see that the distribution of phenolic metabolites in pecan was organ‐specific, tannins tend to accumulate in pecan testa with both diverse structures and high contents, while flavonols tend to accumulate in organs such as branch, bark, or leaf. Among all organs, testa contained the highest content of phenolics, which might play important roles in protecting pecan kernel from diseases and insects. A massive phenolic metabolites' matrix in different pecan organs was built in this experiment, which should be useful for related researches in the future and help provide a theoretical basis for using these organs as functional foods.

## Graphical abstract

A comprehensive qualification and quantification of phenolic metabolites in eight organs of pecan were conducted for the first time. Ninety‐seven phenolic metabolites were identified, in which twelve were identified for the first time in pecan, and thirty‐three phenolic metabolites were quantified. The distribution of phenolic metabolites in pecan were organ‐specific, tannins tend to accumulate in pecan testa with both diverse structures and high contents, while flavonols tend to accumulate in organs such as branch, bark, or leaf.

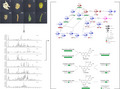

## 1 INTRODUCTION

Pecan [*Carya illinoinensis* (Wangenh.) K. Koch] is an important woody nut crop and had raised great attentions these years for its high phenolic contents (Zhang, Peng, & Li, 2015). It is reported that tree nuts are among the best sources of natural antioxidants and the content of antioxidants in pecan nut ranked as the highest of tree nuts (Wu et al., 2004). Phenolics in pecan kernel were reported to have excellent antioxidant capacities (Biomhoff, Carlsen, Anderson, & Jacobs, 2006; de la Rosa et al., 2014; Prado et al., 2014; Flores‐Cordova et al., 2017; Hilbig, Alves, et al., 2018; Jia et al., 2018; Robbins, Gong, Wells, Greenspan, & Pegg, 2015; de la Rosa, Alvarez‐Parrilla, & Shahidi, 2011; Villarreal‐Lozoya, Lombardini, & Cisneros‐Zevallos, 2007), daily consuming of pecan nut is good to chronic diseases like inflammatory (Robbins, Greenspan, & Pegg, 2016), hyperlipoidemia (Domínguez‐Avila et al., 2015), and can protect human brain, blood, and liver from the damage of oxidant (Domínguez‐Avila et al., 2015; Müller et al., 2013; Reckziegel et al., 2011). Phenolic metabolites in other organs of pecan trees also play critical roles in the defense of pathogens, injuries, and environment stresses. Such as metabolites in pecan leaves might play important roles in the resistant of scab (Lei et al., 2018).

The most important part of pecan is by no means the edible part of the fruit‐the kernel. Pecan fruit consists of pericarp and the seed (nut). The seed includes the inner flesh kernel and the brown pellicle wrapped around the flesh which was called testa. The epicarp and mesocarp were undivided in pecan and together they formed the shuck, while the shell is the endocarp. Besides kernel, other organs of pecan trees also have different values and can be used. Pecan shell is the by‐product of food industry. Pecan shells aqueous extract can protect mice from oxidative damage induced by cigarette smoke exposure and reduced the locomotor activity and anxiety symptoms induced by smoking withdrawal (Reckziegel et al., 2011), it also has hepatoprotective activity against ethanol‐induced liver damage (Müller et al., 2013). The oxidative properties of margarines supplemented with pecan nutshell extracts, rosemary extract, and butylated hydroxytoluene (BHT) were investigated, while pecan nutshell extracts had equally effects with the other two antioxidants and may be considered as a natural product replacement for the synthetic antioxidant BHT (Engler Ribeiro, de Britto Policarpi, Dal Bo, Barbetta, & Block, 2017). The antiproliferative and antitumor activity of pecan shells and their relationship with phenolics were also investigated (de la Rosa et al., 2014; Hilbig, Policarpi, et al., 2018). Chopped pecan shells were used for making tea in Brazil and were thought to have the diuretic and digestive effects (Prado et al., 2014; Engler Ribeiro et al., 2017). Flavonols in pecan bark and leaf were reported to have antidiabetic and hepatoprotective actives (Abdallah, Salama, Abd‐elrahman, & El‐Maraghy, 2011; Gad, Ayoub, & Al‐Azizi, 2007). Many pecan organs besides kernel have the potential of being used in food or health food industry. The bioactives of phenolics in different pecan organs had attracted interests and had been investigated, but the composition and the distribution of these phenolic constituents in most pecan organs were still unclear.

Similar to walnut, pecan also belongs to waste‐heavy materials for that about 70% of the fruit weights are shells and shucks (Han et al., 2018). Harvesting pecan nuts produced a lot of shucks, while cracking pecan nuts produced huge amount of shells. Pecan trees tend to produce excessive male inflorescence, which easily led to the over consume of tree nutrition. Remove over‐bearing male inflorescence can protect the tree and will produce by‐products. Pruning and grafting will also produce by‐products such as leaves, branches, and sometimes barks. Active phenolics were contained in these by‐products, but their compositions were not well understood. A comprehensive metabolic investigation of phenolics in various organs of pecan is needed for better utilization of each part. On the other side, the distribution of secondary metabolites in plants is organ‐specific. They usually share the same upstream metabolic pathway. And then, due to the differences in enzymes activity or type of each organ, these metabolites tend to have different synthetic and catabolic rates or have different chemical modification reactions such as methylation or glycosylation, which led to diverse structures to play diverse functions. So, a comprehensive metabolic investigation of phenolics in different pecan organs is also needed for better understanding of the organ‐specific distribution of these active metabolites in pecan.

By far, the phenolics in pecan were mostly studied in kernels (Gong & Pegg, 2017; Jia et al., 2018; Robbins et al., 2015; Robbins, Ma, Wells, Greenspan, & Pegg, 2014; de la Rosa et al., 2011), while studies in shell (Hilbig, Alves, et al., 2018), leaf (Gad et al., 2007; Ishak, Ahmed, Abd‐Alla, & Saleh, 1980; Lei et al., 2018), and bark (Abdallah et al., 2011) were much less. There is no report of the rest of the organs. The ultra‐high‐performance liquid chromatography coupled with hybrid linear ion trap and Orbitrap mass spectrometer (UHPLC‐LTQ‐Orbitrap MS^n^) is the newest mass technic which has many advantages such as high sensitives and accuracies, while using ultra‐high‐performance liquid chromatography coupled with triple quadrupole mass spectrometer (UHPLC‐QQQ‐MS^n^) can get a better result of quantity. In this paper, we used these methods of both rapid identification and accuracy quantification to get a comprehensive survey of the distribution of phenolic metabolites in various pecan organs.

## 2 MATERIALS AND METHODS

### 2.1 Materials and chemicals

Eight different organs of pecan, including kernel (without testa), testa, shell, shuck, leaf, branch, bark, and flower (male inflorescence), were collected in October 2018 (except the flower were in May 2019) at the scientific orchards of the Institute of Botany, Jiangsu Province and Chinese Academy of Sciences. Samples were collected from healthy adult trees of cultivar “Pawnee.” Six biological replicates were prepared for each organ, and each biological replicate contained samples from three trees. After transported back to the laboratory, the testa was peeled manually from the surface of kernels and was stored at −70°C, so as samples of other parts. Before use, all samples were powdered and homogenized in liquid nitrogen with mortars and pestles.

Menthol, acetic acid, *n*‐hexane, acetonitrile (HPLC grade), and formic acid were purchased from ANPEL Laboratory Technologies. Standard reference compounds, including (+)‐catechin, ellagic acid, and quercetin, were purchased from Nanjing Spring & Autumn Biological CO., Ltd.

### 2.2 Sample extraction

Samples were extracted according to the methods of Regueiro et al. (2014) and Robbins et al. (2014) with slight modifications. The whole experiment was carried out in a dark room illuminated by a red light. Pecan samples (500 mg) and 1 ml mixed solution of methanol/water/acetic acid (70/29.5/0.5, v/v/v) were placed together in a 10‐ml centrifuge tube, ultrasonic extracted for 5 min on ice bath, and centrifuged at 8,000 *g* for 10 min at 4°C (Hettich, Andreas Hettich GmbH & Co. KG). The supernatants were transferred into clean centrifuge tubes. Then, the mixed solutions were added again into the tubes with the kernel residues and ultrasonic extracted again with the same method. The supernatants were combined, and 1 ml *n*‐hexane was added, vortexed for 1 min, centrifuged at 8,000 *g* for 5 min at 4°C for the purpose of defat. This defat process was also repeated again. The methanol layers were collected, combined and stored at −20°C until further analysis.

### 2.3 UHPLC‐LTQ‐Orbitrap MS^n^ qualification

The phenolic metabolites were separated through an ACQUITY UPLC HSS T3 column (2.1 mm × 100 mm, 1.8 μm, Waters) on the Dionex Ultimate 3000 UHPLC system (Thermo Scientific) at 35°C. The mobile phases were 0.1% (*v*/*v*) formic acid (A) and acetonitrile (B), conducted as optimized procedure: 0–1 min, 95% A; 1–5 min, 95%–83% A; 5–12 min, 83%–70% A; 12–15 min, 70%–58% A. The flow rate was 0.4 ml/min, and the injection volume was 2 μl. The qualification was performed on the connected LTQ‐Orbitrap Velos mass spectrometer (Thermo Scientific) equipped with an electrospray ionization source (ESI) source. The parameters were set as follows: capillary voltage, 3 kV; source temperature, 120°C; desolvation temperature, 350°C; cone voltage, 50 V; cone gas flow, 50 L/hr; desolvation gas flow, 600 L/hr; Rutin was used as the lock mass. The mass range was *m/z* 100–2,000.

### 2.4 UHPLC‐QQQ‐MS^n^ quantification

The quantification of phenolic metabolites was performed on the Waters Acquity UPLC system (Waters, Corp.) using the same column and chromatographic conditions as used in previous qualification. Phenolic metabolites were quantified on the AB SCIEX Triple Quad 6500 plus (AB SCIEX Corp.) equipped with an ESI source. The MS was conducted under multi reaction monitoring (MRM) mode, and the parameters were set as follows: capillary voltage, 3.5 kV; source temperature, 150°C; desolvation temperature, 400°C; cone voltage, 50 V; cone gas flow, 50 L/hr; desolvation gas flow, 1,000 L/hr; collision gas flow, 0.15 ml/min. The declustering potential and collision energy were set to match the MRM of each marker. The dwell time was automatically set by the MultiQuant software. The raw data were processed with MultiQuant 3.0.2 software.

### 2.5 Total phenolic content

The total phenolic content was measured according to the methods of our previous report (Jia et al., 2018). Briefly, methanol extract (10 μl) was mixed with 2 ml of 7.5% (w/v) sodium carbonate and 2.5 ml of 10% (v/v) Folin–Ciocalteu regent, and put in water bath at 50°C for 15 min in the dark. The reaction solutions were cooled to room temperature, and then, the absorbance was measured at 760 nm using UV spectrophotometer (Shimadzu UV‐2100, Shimadzu Corporation). Ellagic acid was used as standard reference, and the results were expressed as milligrams of ellagic acid equivalents (EAE) per gram of defatted kernel weight (mg EAE g^−1^). The bland solution was made by pure methanol and treated along with samples under the same protocol. All samples were measured in triplicates on three biological replicates.

### 2.6 Antioxidant capacities

#### 2.6.1 2, 2‐Diphenyl‐1‐picrylhydrazyl (DPPH) free radical scavenging assay

The antioxidant capacities were firstly measured by the DPPH free radical scavenging assay according to the methods of previous report (Jia et al., 2018). Briefly, 4 ml of DPPH radical solution (39.43 mg DPPH in 1 L methanol) was mixed with 10 μl of methanol extract and kept in dark for 30 min. Then, absorbance was measured at 515 nm with UV spectrophotometer. The bland solution was made by pure methanol and treated along with samples under the same protocol. All samples were measured in triplicates on three biological replicates. Absorbance of blank was subtracted from each sample. Trolox was used as standard reference, and the results were expressed as μmol trolox equivalents (TE) per gram of defatted kernel weight (μmol TE g^−1^).

#### 2.6.2 2, 2′‐Azino‐bis (3‐ethylbenzothiazoline‐6‐sulphonic acid) diammonium salt (ABTS) free radical scavenging assay

Then, the antioxidant capacities were measured by the ABTS free radical scavenging assay according to previous reports of Salvador, Podestá, Block, and Ferreira (2016) and Prado et al. (2013). Briefly, 38.36 mg of ABTS was dissolved in 10 ml deionized water to get the ABTS˙^+^ solution (7.0 mM). Then, the ABTS˙^+^ solution was mixed with 2.45 mM potassium persulphate solution on the ratio of 1:1 (v/v). The mixture was kept under dark for at least 16 hr and diluted with ethanol to absorbance of 0.70 ± 0.05 at 734 nm with UV spectrophotometer before use. Then, 40 μl methanol extracts of samples were mixed with 2 ml ABTS˙^+^ solution, and the absorbance was measured at 734 nm after rested still for 6 min. The bland solutions were made by pure methanol and treated along with samples under the same protocol. All samples were measured in triplicates on three biological replicates. Absorbance of blank was subtracted from each sample. Trolox was used as standard reference, and the results were expressed as μmol trolox equivalents per gram of defatted kernel weight (μmol TE g^−1^).

### 2.7 Method validation and statistical analysis

Commercial standard compounds were used to validate the linearity, precision, repeatability, and stability of the optimized method. At least six appropriate concentrations of standard solution in duplicate were prepared to obtain the linearity. The LOD (limit of detection) and LOQ (limit of qualification) for each analyte were acquired at a S/N of 3 and 10, respectively. Six injections of mixed standard solutions, including the highest, middle, and lowest concentration of linearity, were used to assess the precision and reproducibility. Six independent replicates were studied for each sample solution. Sample stability was assessed by analyzing it at 0, 2, 4, 8, 12, and 24 hr. The accuracy of the method was performed by adding the corresponding marker compounds with high (120% of the known amounts), middle (same as the known amounts), and low (80% of the known amounts) levels to 0.25 g of samples analyzed previously. Triplicate experiments were performed at each level. The average recoveries were estimated by the formula: recovery (%) = [(amount found − original amount)/amount added] × 100 (Zhang et al., 2019). The MS data were processed by the Xcalibur (version 4.1) software, and PCA was performed by the SPSS (version 18.0) software.

## 3 RESULTS AND DISCUSSION

### 3.1 Method validation

The liquid chromatographic method had been optimized, and the final parameters were chosen under the consideration of both separation resolution and analytical efficiency. Commercial standard compounds were used to verify the optimized analytical method (Table S1). Results showed that the method had good linearity over wide ranges with regression coefficients (*r* ^2^) over .99. The LOD and the LOQ were 0.0009 μg/ml and 0.0029 μg/ml for catechin, 0.0008 μg/ml and 0.0026 μg/ml for ellagic acid, 0.0008 μg/ml and 0.0028 μg/ml for quercetin, respectively. The precision, repeatability, and stability of the method were also studies using standard compounds (Table S2). The low relative standard deviations (RSDs) demonstrated that this method is precise, repeatable, and stable and can be used in the following experiment.

### 3.2 Identification of phenolic metabolites in different pecan organs

**FIGURE 1:**
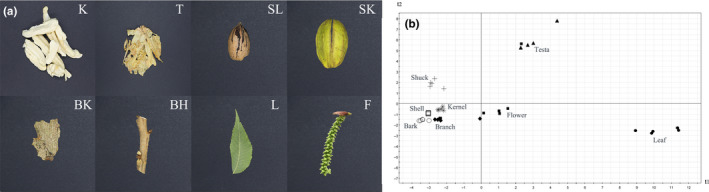
Pecan organs analyzed for phenolic metabolites. (a) K, kernel; T, testa; SL, shell; SK, shuck; BK, bark; BH, branch; L, leaf; F, flower (male inflorescence); (b) Principal component analysis of the phenolic metabolites (performed with peak areas). *n* = 5 except testa (*n* = 4) and flower (*n* = 6)

A total of 97 phenolic metabolites have been identified from 8 pecan organs, including 57 hydrolysable tannins, 19 condensed tannins, 1 complex tannin, 18 flavonols, and 2 other compounds (Figure 1a, Table 1). There were significant differences in the numbers of metabolites identified in different pecan organs (Table 1). The number of phenolic compounds identified from leaves was largest among all organs, followed by testa, while the least phenolic compounds were identified from shells. This result showed the difference in the complexity of phenolic compounds among different pecan organs, similar result can be seen in the principal component analysis (PCA) too (Figure 1b). The samples of testa and leaf were far from the others, which showed the specificity of phenolic compounds in these organs. The samples of kernel, shell, branch, and bark were close to each other, which showed the similarity of composition between them. The phenolic compositions of kernel were quite different from those of testa. Of the 97 phenolic metabolites, 30 were found in all organs, 34 in only one organ, and 21 in only two organs (Table 1).

**TABLE 1:** Phenolic metabolites tentatively identified in pecan organs^†^

Peak No.	RT (min)	Compound identification	Formula	[M−H] (*m/z*)	MS/MS fragments (*m/z*)	Δ*m* (ppm)	K	T	SL	SK	BK	BH	L	F
1	0.72	HHDP‐glucose	C_20_H_18_O_14_	481.0641	301–275	−4.78	√	√	√	√	√	√	√	√
2	0.95	HHDP‐glucose	C_20_H_18_O_14_	481.0632	301–275	−2.91	√	√	√	√	√	√	√	√
3	1.10	HHDP‐glucose	C_20_H_18_O_14_	481.0628	301–275	−2.08	√	√	√	√	√	√	√	√
4	1.31	Gallic acid hexoside	C_13_H_16_O_10_	331.0647	169	5.44							√	
5	1.42	Gallic acid hexoside	C_13_H_16_O_10_	331.0647	169	5.44								√
6	1.43	Pterocarinin B^‡^	C_39_H_32_O_26_	915.1122	871–781–733–569–301	−1.97						√	√	
7	1.6	Valoneoyl‐glucose	C_27_H_22_O_19_	649.0694	605–481–301	−2.62							√	
8	1.72	Valoneoyl‐glucose	C_27_H_22_O_19_	649.0694	605–481–301	−2.62	√	√	√	√		√	√	√
9	2.47	Pedunculagin/casuariin isomer	C_34_H_24_O_22_	783.0695	481–301	−1.79						√	√	
10	2.57	Procyanidin trimer (C1)	C_45_H_38_O_18_	865.1987	739–713–695–577–575–287	−0.81		√		√	√	√		
11	3.00	Azaleatin rhamnoside	C_22_H_22_O_11_	461.1101	315	−3.69					√	√		
12	3.02	Pedunculagin/casuariin isomer	C_34_H_24_O_22_	783.0690	481–301	−1.15	√	√	√	√	√	√	√	√
13	3.21	Pedunculagin/casuariin isomer	C_34_H_24_O_22_	783.0689	481–301	−1.02	√	√				√	√	
14	3.69	1 (E)C and 1 (E)GC B linkage	C_30_H_26_O_13_	593.1309	467–425–407–289	−2.36	√	√	√	√	√	√	√	√
15	3.87	Pedunculagin/casuariin isomer	C_34_H_24_O_22_	783.0630	481–301	6.51								√
16	3.92	Neochlorogenic acid^‡^	C_16_H_18_O_9_	353.0885	191–179–135	−3.40							√	
17	4.05	Glansrin C^‡^	C_41_H_26_O_26_	933.0653	631–451–301	−2.04		√						
18	4.25	Pedunculagin/casuariin isomer	C_34_H_24_O_22_	783.0690	481–301	−1.15	√	√	√	√	√	√	√	√
19	4.53	Praecoxin A/platycariin isomer	C_41_H_28_O_27_	951.0789	907–783–481–301	−5.15	√	√	√	√	√	√	√	√
20	4.73	Procyanidin dimer B linkage	C_30_H_26_O_12_	577.1364	451–425–407–289	−3.12	√	√	√	√	√	√	√	√
21	4.81	Procyanidin dimer B linkage	C_30_H_26_O_12_	577.1364	451–425–407–289	−3.12	√	√				√		
22	4.85	Praecoxin A/platycariin isomer	C_41_H_28_O_27_	951.0759	907–783–481–301	−2.00	√	√	√	√	√	√	√	√
23	4.92	Pedunculagin/casuariin isomer	C_34_H_24_O_22_	783.0630	481–301	6.51								√
24	5.01	Procyanidin dimer B linkage	C_30_H_26_O_12_	577.1372	451–425–407–289	−4.51	√	√	√	√	√			√
25	5.12	Tellimagrandin I	C_34_H_26_O_22_	785.0854	633–483–301	−2.04	√	√	√	√	√	√	√	√
26	5.25	Pterocarinin A^‡^	C_46_H_36_O_30_	1,067.1257	1,023–933–765–377–301	−4.12		√					√	
27	5.28	(+)‐Catechin	C_15_H_14_O_6_	289.0726	245–175	−4.84	√	√	√	√	√	√	√	√
28	5.36	Stenophyllanin A/B isomer^‡^	C_56_H_40_O_31_	1,207.1541	917–873–855	−5.47		√					√	
29	5.37	Pterocarinin A^‡^	C_46_H_36_O_30_	1,067.1268	1,023–933–765–377–301	−5.15							√	
30	5.49	Procyanidin trimer (C1)	C_45_H_38_O_18_	865.2026	739–713–695–577–575–287	−5.32	√	√		√	√	√	√	
31	5.53	Strictinin/isostrictinin isomer	C_27_H_22_O_18_	633.0762	481–301	−5.37						√	√	
32	5.53	Tris‐O‐degalloyl rugosin F isomer^‡^	C_61_H_44_O_40_	707.0638[M−2H]^2−^ (MW 1,415)	1,114–934–783–633–481–301	−4.95	√	√						
33	5.57	Cinnamtannin A2	C_60_H_50_O_24_	1,153.2637	865–713–577–575–413	−1.99		√		√	√	√	√	
34	5.72	Casuarinin/casuarictin isomer^‡^	C_41_H_28_O_26_	935.0845	783–633–481–301	−5.77	√	√	√	√	√	√	√	√
35	5.76	Strictinin/isostrictinin isomer	C_27_H_22_O_18_	633.0682	481–301	7.27								√
36	5.93	Cinnamtannin A2	C_60_H_50_O_24_	1,153.2651	865–713–577–575–413	−3.21		√					√	
37	5.97	Praecoxin A/platycariin isomer	C_41_H_28_O_27_	951.0782	907–783–481–301	−4.42	√	√	√	√	√	√	√	√
38	6.02	Tellimagrandin I	C_34_H_26_O_22_	785.0865	633–483–301	−3.44		√					√	
39	6.06	Brevifolin carboxylic acid	C_13_H_8_O_8_	291.0155	247	−4.81	√	√	√			√		
40	6.18	Glansrin C^‡^	C_41_H_26_O_26_	933.0671	631–451–301	−3.97		√						
41	6.26	Stenophyllanin A/B isomer^‡^	C_56_H_40_O_31_	1,207.1397	917–873–855	6.46	√	√	√	√	√	√	√	√
42	6.33	Valoneic acid dilactone	C_21_H_10_O_13_	469.0069	425–301	−5.54							√	
43	6.38	Procyanidin dimer B linkage	C_30_H_26_O_12_	577.1357	451–425–407–289	−1.91	√	√	√	√	√	√	√	√
44	6.45	Valoneic acid dilactone	C_21_H_10_O_13_	469.0067	425–301	−5.12							√	
45	6.54	Reginin A/D isomer^‡^	C_75_H_50_O_48_	858.0762[M−2H]^2−^ (MW 1717)	933–783–633–481–301	−6.81		√						
46	6.65	Procyanidin trimer (C1)	C_45_H_38_O_18_	865.1995	739–713–695–577–575–287	−1.73						√		
47	6.66	Cinnamtannin A2	C_60_H_50_O_24_	1,153.2667	865–713–577–575–413	−4.60		√		√		√		
48	6.69	Glansrin C^‡^	C_41_H_26_O_26_	933.0674	631–451–301	−4.29							√	
49	6.74	Reginin A/D isomer^‡^	C_75_H_50_O_48_	858.0759[M−2H]^2−^ (MW 1717)	933–783–633–481–301	−5.94		√						
50	6.98	Casuarinin/casuarictin isomer^‡^	C_41_H_28_O_26_	935.0723	783–633–481–301	7.27								√
51	7.03	Rugosin C/glansrin A isomer^‡^	C_48_H_32_O_31_	1,103.0906	1,059–935–757–633–301	−5.17		√					√	
52	7.04	Procyanidin dimer B linkage	C_30_H_26_O_12_	577.1360	451–425–407–289	−2.43				√		√		
53	7.17	Cinnamtannin A3	C_75_H_62_O_30_	1,441.3262	1,289–1,153–1,151–865–577–575	−0.97				√				
54	7.21	Glansrin C^‡^	C_41_H_26_O_26_	933.0576	631–451–301	6.22								√
55	7.23	Pedunculagin/casuariin isomer	C_34_H_24_O_22_	783.0722	481–301	−5.24	√	√						
56	7.47	Azaleatin galactose	C_22_H_22_O_12_	477.1046	315	−2.72						√		
57	7.52	Quercetin galloyl hexoside	C_28_H_24_O_16_	615.0945	463–301	6.67								√
58	7.56	Rugosin C/glansrin A isomer^‡^	C_48_H_32_O_31_	1,103.0907	1,059–935–757–633–301	−5.26							√	
59	7.60	Ellagic acid pentose	C_19_H_14_O_12_	433.0428	301	−4.85	√	√	√	√	√	√	√	√
60	7.60	Azaleatin glucoside	C_22_H_22_O_12_	477.1049	315	−3.35					√	√		
61	7.72	Quercetin galloyl hexoside	C_28_H_24_O_16_	615.1016	463–301	−4.88						√	√	
62	7.78	Cinnamtannin A3	C_75_H_62_O_30_	1,441.3235	1,289–1,153–1,151–865–577–575	0.90				√				
63	7.79	Azaleatin arabinoside	C_21_H_20_O_11_	447.0946	315	−4.25					√	√		
64	7.81	Casuarinin/casuarictin isomer^‡^	C_41_H_28_O_26_	935.0841	783–633–481–301	−5.35		√						
65	7.91	Azaleatin rhamnoside	C_22_H_22_O_11_	461.1106	315	−4.77	√	√	√			√	√	√
66	7.92	Quercetin galloyl hexoside	C_28_H_24_O_16_	615.1019	463–301	−5.36							√	
67	7.93	Casuarinin/casuarictin isomer^‡^	C_41_H_28_O_26_	935.0767	783–633–481–301	2.57	√							
68	7.96	Quercetin galloyl hexoside	C_28_H_24_O_16_	615.1019	463–301	−5.36							√	
69	7.97	Methyl ellagic acid galactose	C_21_H_18_O_13_	477.0682	315–300	−2.72	√							
70	7.97	Heterophylliin D^‡^	C_82_H_54_O_52_	934.0767[M−2H]^2−^ (MW 1869)	1,568–1,085–783–633–451–301	−7.12		√	√					
71	8.00	Cinnamtannin A3	C_75_H_62_O_30_	1,441.322	1,289–1,153–1,151–865–577–575	1.94				√				
72	8.11	Procyanidin dimer A linkage	C_30_H_24_O_12_	575.1202	449–423–407–289	−2.09						√		
73	8.19	Quercetin hexoside	C_21_H_20_O_12_	463.0900	301	−4.97							√	√
74	8.22	Ellagic acid	C_14_H_6_O_8_	300.9998	257–229	−4.65	√	√	√	√	√	√	√	√
75	8.31	Quercetin hexoside	C_21_H_20_O_12_	463.0897	301	−4.32	√	√	√	√	√	√	√	√
76	8.39	Methyl ellagic acid glucose	C_21_H_18_O_13_	477.0690	315–300	−4.40	√	√	√	√	√	√	√	√
77	8.46	Quercetin hexoside	C_21_H_20_O_12_	463.0891	301	−3.02				√		√	√	
78	8.64	Eucalbanin A/cornusiin B isomer^‡^	C_48_H_30_O_30_	1,085.0780	933–783–633–451–301	−3.32		√						√
79	8.79	(Epi) catechin gallate	C_22_H_18_O_10_	441.0837	331–289–271–169	−3.40		√						
80	8.93	Caryatin sulfate	C_17_H_14_O_10_S	409.0248	329	−4.65					√	√		
81	9.07	Quercetin pentose	C_20_H_18_O_11_	433.0792	301	−4.85	√	√	√	√	√	√	√	√
82	9.15	Eucalbanin A/cornusiin B isomer^‡^	C_48_H_30_O_30_	1,085.0781	933–783–633–451–301	−3.41							√	
83	9.28	Methyl ellagic acid pentose	C_20_H_16_O_12_	447.0581	315–300–198–161	−3.80	√	√	√	√	√	√	√	√
84	9.35	Quercetin pentose	C_20_H_18_O_11_	433.0784	301	−3.00	√	√	√	√	√	√	√	√
85	9.46	Ellagic acid rhamnoside	C_20_H_16_O_12_	447.0579	301	−3.36	√	√	√	√	√	√	√	√
86	9.58	Quercetin rhamnoside	C_21_H_20_O_11_	447.0949	301	−4.92	√	√	√	√	√	√	√	√
87	9.70	Caryatin	C_17_H_14_O_7_	329.0671	314–301	−3.04	√	√	√	√	√	√	√	√
88	9.74	Ellagic acid galloyl pentose	C_26_H_18_O_16_	585.0536	433–301	−3.25		√					√	
89	9.95	Procyanidin dimer A linkage	C_30_H_24_O_12_	575.1204	449–423–407–289	−2.43		√			√	√		
90	10.15	Methyl ellagic acid	C_15_H_8_O_8_	315.0148	301	−2.22	√	√	√	√	√	√	√	√
91	10.34	Ellagic acid galloyl pentose	C_26_H_18_O_16_	585.0545	433–301	−4.79							√	
92	10.87	Dimethyl ellagic acid rhamnoside^‡^	C_22_H_20_O_12_	475.0888	329	−2.32	√	√	√	√	√	√	√	√
93	11.59	Methyl ellagic acid galloyl pentose	C_27_H_20_O_16_	599.0689	447–433–315–301	−2.67		√					√	
94	11.78	Caryatin methyl ether	C_18_H_16_O_7_	343.0830	329	−3.50					√			
95	11.93	Methyl ellagic acid galloyl pentose	C_27_H_20_O_16_	599.0687	447–433–315–301	−2.34	√	√	√	√	√	√	√	√
96	12.17	Methyl ellagic acid galloyl pentose	C_27_H_20_O_16_	599.0687	447–433–315–301	−2.34	√	√	√	√	√	√	√	√
97	12.55	Methyl ellagic acid galloyl pentose	C_27_H_20_O_16_	599.0685	447–433–315–301	−2.00	√	√	√	√	√	√	√	√
Total number							41	58	35	41	40	53	60	42

^†^ K, kernel; T, testa; SL, shell; SK, shuck; BK, bark; BH, branch; L, leaf; F, flower (male inflorescence); E, epi; C, catechins; GC, gallocatechin.

^‡^ These compounds were identified for the first time in pecan.

#### 3.2.1 Identification of hydrolysable tannins and their distribution

**FIGURE 2:**
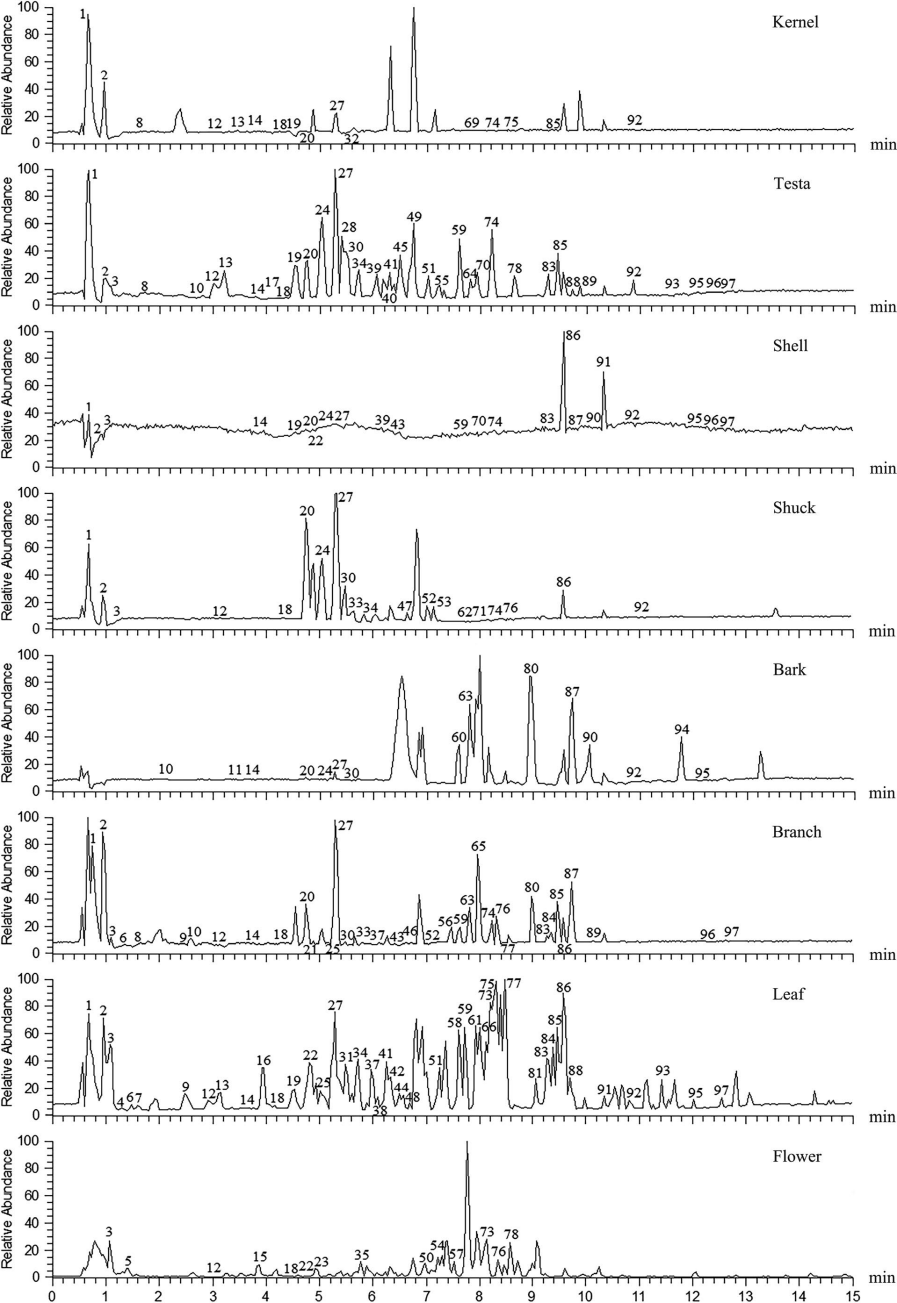
UHPLC‐LTQ‐Orbitrap MS total ion current chromatograms in negative ion mode of eight pecan organs

The major phenolic compounds identified were hydrolysable tannins, accounting for about two‐thirds of the total number (Figure 2). Peak 74 with molecular ion at *m/z* 300.9998 ([M−H]^−^) was identified as ellagic acid by comparing its fragments with previous reports and its *t_R_* value with standard compound. Ellagic acid pentose (peak 59) and ellagic acid rhamnoside (peak 85) can be assured by the fragment ion at *m/z* 301, which represent the ellagic acid aglycone, and a loss of 132 Da (pentose) and 146 Da (deoxyhexose) in the MS^2^ spectrum. Methyl ellagic acid derivatives (peaks 69, 76, 83, and 90) can be recognized by the ions at *m/z* 315 and loss of a methyl group, while two sequential losses of methyl groups gave evidence of peak 92 to be dimethyl ellagic acid derivative. According to the fragment pattern, peak 92 was assigned as dimethyl ellagic acid rhamnoside, while this is the first report of this compound in pecan. Peak 83 had the same molecular weight (MW) of 447 with peak 85, but their fragment patterns were different. Peak 83 showed fragment ions at *m/z* 315 ([M−H−132]^−^, loss of pentose) and 300 ([M−H−132–15]^−^, loss of pentose and methyl group), indicated that it was methyl ellagic acid pentose. All these compounds except peak 69 were found in all organs.

Hexahydroxydiphenoyl (HHDP) group is a basic structure of ellagitannins. HHDP‐glucose (peaks 1, 2, and 3, [M−H]^−^, *m/z* 481) were identified in all organs and they eluted really fast (Figure 2). Seven metabolites (peaks 9, 12, 13, 15, 18, 23, and 55) were identified as pedunculagin/casuariin isomer (bis‐HHDP‐glucose) according to the corresponded precursor ion at *m/z* 783 ([M−H]^−^), with its fragmentation ions at 481 ([M−H−302]^−^, loss of HHDP) and 301 ([M−H−482]^−^, loss of HHDP‐glucose). These metabolites also distributed in all organs. Peak 6 ([M−H]^−^, *m/z* 915) was tentatively assigned as pterocarinin B, which possessed one more pentose than pedunculagin/casuariin isomer. This ellagitannin had been found in *Pterocarya stenoptera* and walnut before (Fukuda, Ito, & Yoshida, 2003; Nonaka, Ishimaru, Azuma, Ishimatsu, & Nishioka, 1989); it was found in samples of leaves and branches in this experiment, this is the first report in pecan.

Gallotannins had also been found in the earlier eluents. The deprotonated molecular ions at *m/z* 331.0647 and the fragments at 169 indicated that peaks 4 and 5 contained gallic acid and hexose moieties. Peak 4 had only been detected in leaf sample, while peak 5 only in male inflorescence sample.

More hydrolyzed tannins identified in pecan were more complicated and mixed with both HHDP and galloyl groups. Peaks 42 and 44 ([M−H]^−^, *m/z* 469) were identified as valoneic acid dilactone, which contained one ellagic acid moiety (*m/z* 301 in MS^2^ spectrum) and one gallic acid moiety (loss of 168 in MS^2^ spectrum) conjugated with C‐C bond. They were found only in pecan leaves in this experiment.

Metabolites detected at *m/z* 585 (peaks 88 and 91) and *m/z* 599 (peaks 93, 95, 96, and 97) were assigned as ellagic acid galloyl pentose and methyl ellagic acid galloyl pentose, respectively.

On the basis of HHDP‐glucose (*m/z* 481), metabolites with one to three galloyl substituents were found at *m/z* 633 (peaks 31 and 35), 785 (peaks 25 and 38), and 935 (peaks 34, 50, 64, and 67) and were assigned as strictinin/isostrictinin isomer, tellimagrandin I, and casuarinin/casuarictin isomer. This is the first report of casuarinin/casuarictin isomer in pecan. Casuarinin and previous mentioned casuariin were first isolated from *Casuarina stricta* (Okuda, Yoshida, Ashida, & Yazaki, 1982), and they were widely distributed in many species including *Juglandaceae* families (Okuda, Yoshida, Hatano, Yazaki, & Ashida, 1980) and were building blocks of C‐glycosidic tannins (Okuda, Yoshida, Hatano, & Ito, 2009).

On the basis of pedunculagin/casuariin isomer (bis‐HHDP‐glucose, *m/z* 783), metabolites with one and two galloyl substituents were found at *m/z* 933 (peaks 17, 40, 48, and 54) and 1,085 (peaks 78 and 82) and were assigned as glansrin C and eucalbanin A/cornusiin B isomer. Glansrin C and eucalbanin A/cornusiin B isomer had been detected from walnut before (Regueiro et al., 2014), and this is the first report of these two tannins in pecan. Peaks 19, 22, and 37 produced [M−H]^−^ ions at *m/z* 951 and had similar series of ions at *m/z* 783, 633, 481, and 301 in MS^2^ spectrum, indicated they contained an extra group of gallic acid and they were assigned as praecoxin A/platycariin isomer.

One more galloyl group linked to the HHDP unit will form valoneoyl, tergalloyl, or macaranoyl groups (Okuda et al., 2009). Peaks 7 and 8 had the deprotonated molecular ion ([M−H]^−^) at *m/z* 649.0694, with its fragments at *m/z* 605 ([M−H−44]^−^, loss of carboxyl), 481([M−H−168]^−^, loss of gallic acid) and 301, were identified as valoneoyl‐glucose, though tergalloyl‐glucose or macaranoyl‐glucose were also possible. Through analysis of the fragmentation patterns, peaks 51 and 58 ([M−H]^−^, 1,103) can be assigned as rugosin C/glansrin A isomer which contained one more valoneoyl group on the basis structure of strictinin/isostrictinin isomer. This is the first report of rugosin C/glansrin A isomer in pecan.

Peaks 26 and 29 generated [M−H]^−^ ions at *m/z* 1,067 and gave fragment ions at *m/z* 933 ([M−H−134]^−^, loss of pentose) showed the existence of an extra pentose on the basis of glansrin C. Combined this information with literature reports, they were determined to be pterocarinin A. This compound had been found in walnut before (Regueiro et al., 2014), while this is the first report in pecan.

**FIGURE 3:**
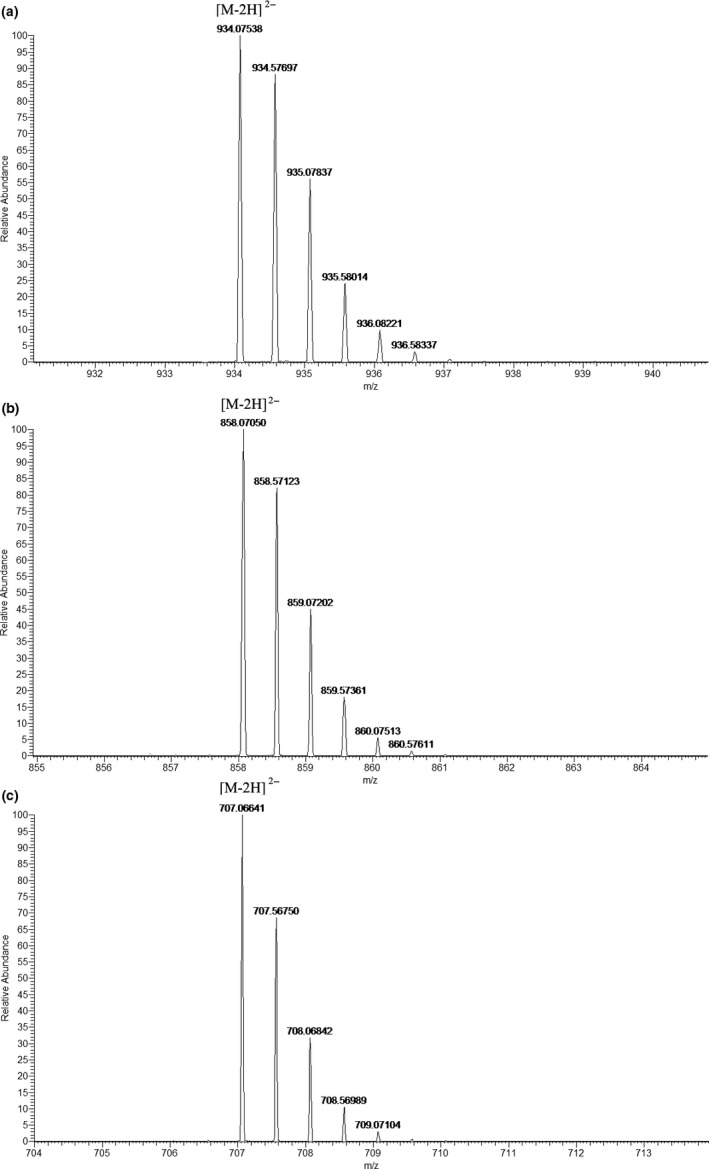
The doubly charged ions of heterophylliin D (a), reginin A/D isomer (b), and tris‐O‐degalloyl rugosin F isomer (c)

**FIGURE 4:**
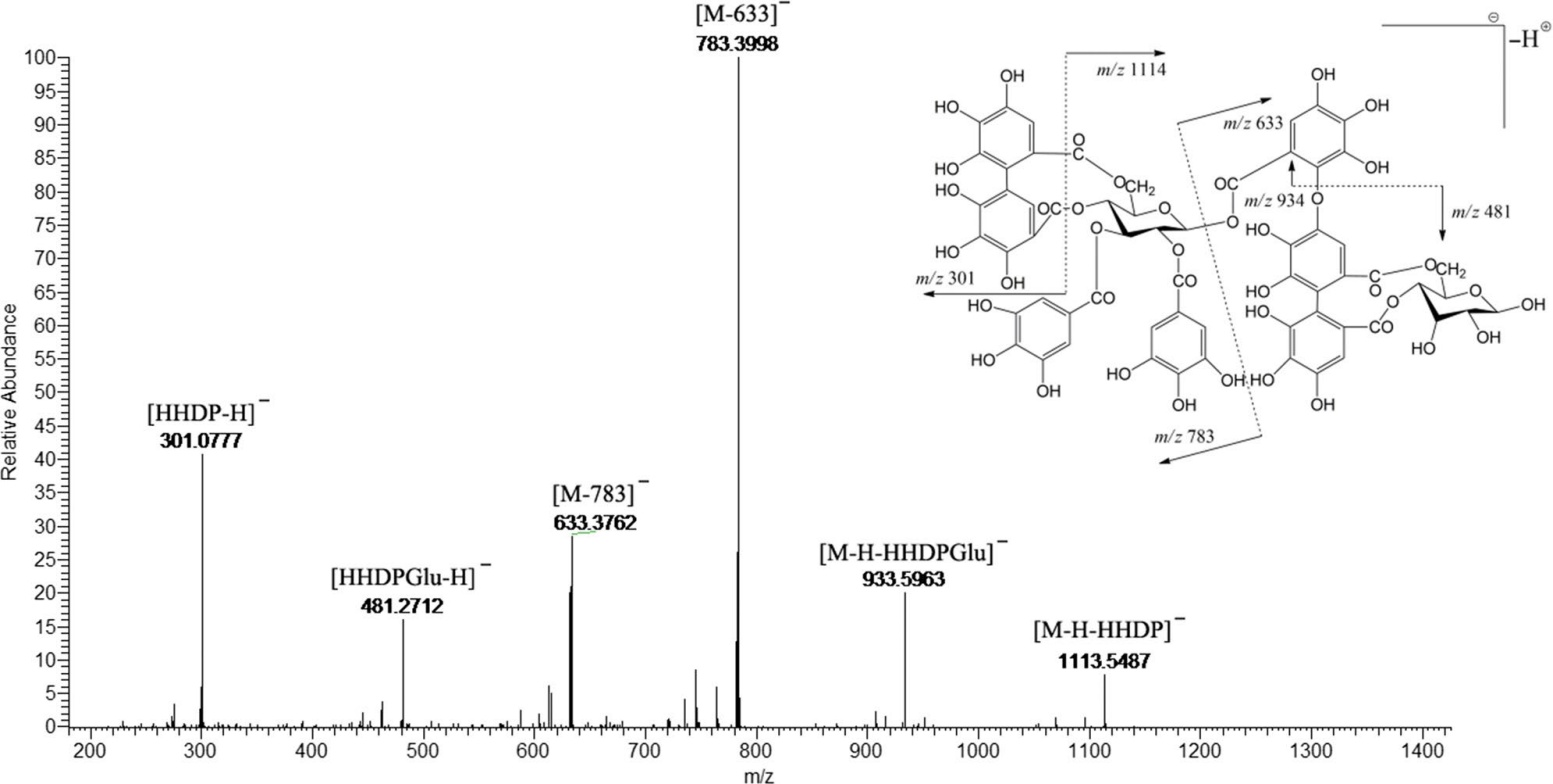
MS/MS fragmentation spectrum of a tris‐O‐degalloyl rugosin F isomer

**FIGURE 5:**
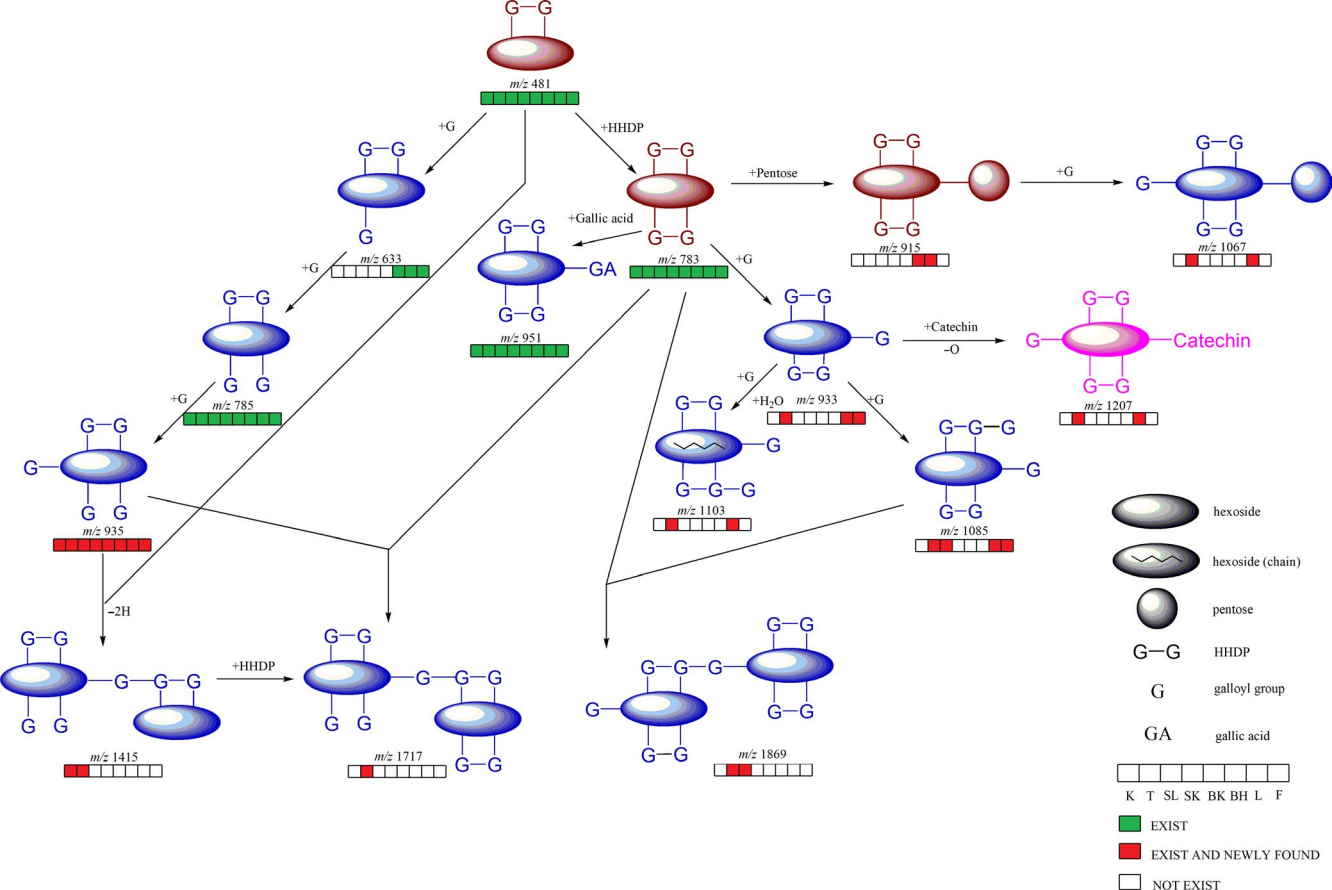
Deduced metabolic pathway of part of the hydrolysable tannins in pecan organs. Different colors of metabolites represented different structure categories: brown, ellagitannins; blue, hydrolysable tannins mixed with both HHDP and galloyl groups; pink, complex tannin. K, kernel; T, testa; SL, shell; SK, shuck; BK, bark; BH, branch; L, leaf; F, flower (male inflorescence)

Several ellagitannins were identified through their doubly charged ions ([M−H]^2−^) which were confirmed by the isotopic peaks (Figure 3), such as peak 32 showed [M−2H]^2−^ ion at *m/z* 707.0638 indicated its MW of 1,415. Meanwhile, the fragment ions at *m/z* 1,114, 934, 783, 633, 481, and 301 in MS^2^ spectrum showed that it was composed of two ellagitannins, casuarinin/casuarictin isomer, and HHDP‐glucose (Figure 4). This ellagitannin was identified as tris‐O‐degalloyl rugosin F isomer and was found in pecan testa and kernel in this experiment. Peaks 45 and 49 generated doubly charged ions at *m/z* 858.0762 [M−2H]^2−^ and similar fragment ions at *m/z* 933, 783, 633, 481, and 301. This meant that these ellagitannins had a MW of 1,717 and were composed of two structures, tellimagrandin I and glansrin C, so they were identified as reginin A/D isomer. This ellagitannin was found only in pecan testa in this experiment. The MW of peak 70 is the highest among the peaks we identified. Its doubly charged ion was found at *m/z* 934.0767 [M−2H]^2−^, indicating that its MW was 1,869. Through the analysis of fragment ions, it was found that this ellagitannin was composed of tellimagrandin I and eucalbanin A/cornusiin B isomer, so it was identified as heterophylliin D. All these high MW ellagitannins were reported the first time in pecan. Through this study, the tannin profile and each route of tannin metabolic pathway of pecan had been expanded (Figure 5), which laid a foundation for future researches and uses.

#### 3.2.2 Identification of condensed tannins and their distribution

A series of condensed tannins were identified in these organs. Peak 27 (*t_R_* = 5.28 min) was assured as (+)‐catechin, the basic building blocks of CTs, by the molecular ion at *m/z* 289.0726 and comparing with commercial standard. This compound was found in all organs. The (epi)catechin gallate (peak 79) was only found in testa at 8.79 min in minimum quantity and cannot be found in other organs. There were two kinds of linkage between the building blocks of procyanidins, in which B type is more common because of less space resistant. Five metabolites (peaks 20, 21, 24, 43, and 52) were identified as procyanidin dimer B linkage (PD2B) according to the deprotonated molecular ion at *m/z* 577 ([M−H]^−^) and the fragment ion at *m/z* 289 ([M−H−288, loss of a catechin moiety]^−^). Peaks 20 and 43 were found in all organs. Two metabolites (peaks 72 and 89) were identified as procyanidin dimer A linkage according to the molecular ion at *m/z* 575 ([M−H]^−^), two hydrogen atoms less than PD2B. They can only be found in branch, testa, and bark. Peak 14 was also procyanidin dimer but contained one (epi)catechin and one (epi)gallocatechin. Procyanidin trimer (peaks 10, 30, and 46), tetramer (peaks 33, 36, and 47), and pentamer (peaks 53, 62, and 71) were also identified, in which pentamer were only found in shuck.

#### 3.2.3 Identification of flavonols and their distribution

All flavonoid metabolites identified in this experiment were flavonols, and most of them were eluted in the later part of elutes. There were three types of flavonol aglycones, quercetin, azaleatin, and caryatin, which contained their identical fragment ions at *m/z* 301, 315, and 329 in the MS^2^ spectrum, respectively. One tricky problem about flavonol identification in pecan is they have similar MW with ellagic acid, methyl ellagic acid, and dimethyl ellagic acid derivatives. Luckily, the high‐accuracy MW can solve these problems.

For example, the high‐accuracy MW of peaks 56 and 60 ([M−H]^−^ 477.10) can help us discriminated them from peaks 69 and 76 ([M−H]^−^ 477.06), their structures were determined as azaleatin hexoside instead of methyl ellagic acid hexoside according the MW and the fragment ions at *m/z* 315. It is reported that the galactosides eluted earlier than its corresponding glucosides (Abad‐García, Berrueta, Garmón‐Lobato, Gallo, & Vicente, 2009), so peak 56 was tentatively assigned as azaleatin galactose, and peak 60 was tentatively assigned as azaleatin glucoside. Ishak et al. had isolated a series of azaleatin glycosides from pecan branch including glucoside, rhamnoside, arabinoside, diglucoside, and rutinoside (Ishak et al., 1980), and their structures were elucidated with NMR method, so this is the first report of azaleatin galactose in pecan, and this is also the first report of azaleatin glucoside in pecan bark.

The high‐accuracy MW also helped us to identify peak 81 (experimental MW 433.0792) and 84 (experimental MW 433.0784) to be quercetin pentose (C_20_H_18_O_11_, theoretical MW 433.0771) instead of ellagic acid pentose (peak 59, C_19_H_14_O_12_, theoretical MW 433.0407). Similarly, peaks 73, 75, and 77 were assigned as quercetin hexosides (C_21_H_20_O_12_, theoretical MW 463.0877) according to their high‐accuracy MW 463.09, instead of ellagic acid hexoside (C_20_H_16_O_13_, theoretical MW 463.0513). Peaks 57, 61, 66, and 68 ([M−H]^−^, 615) were assigned as quercetin galloyl hexoside instead of ellagic acid galloyl hexoside. A series of quercetin glycosides had been isolated from pecan leaves and elucidated with NMR method, including glucoside, rhamnoside, arabinoside, galactoside, and galloyl galactoside (Abdallah et al., 2011; Gad et al., 2007; Ishak et al., 1980).

The molecular ion at *m/z* 329 and the fragment ions at *m/z* 314 and 301 of peak 87 were consistent with the dimethyl ellagic acid (C_16_H_10_O_8_, theoretical MW 329.0297) which had been reported in pecan kernel, but the experimental MW of 329.0671 denied this deduction, instead it complied with caryatin (C_17_H_14_O_7_, theoretical MW 329.0661).

The high‐accuracy MW of both peaks 63 and 86 were 447.09, suggested their molecular formula to be C_21_H_20_O_11_. Combined with information of fragment ion at *m/z* 315, peak 63 can be tentatively assigned as azaleatin arabinoside, which had been previously reported in pecan branches (Ishak et al., 1980). Peak 86 had the same molecular formula but different structure, the fragment ion at *m/z* 301 suggested it contained a quercetin aglycone and a rhamnoside; therefore, it was assigned as quercetin rhamnoside, which had been reported in pecan leaves (Gad et al., 2007). Azaleatin arabinoside had been found in branch and bark, while quercetin rhamnoside had been found in all organs. So, there were four peaks (peaks 63, 83, 85, and 86) had similar MW of 447, but their structures were all different with each other, which were azaleatin arabinoside, methyl ellagic acid pentose, ellagic acid rhamnoside, and quercetin rhamnoside. With powerful tools of both the high‐accuracy MW and the fragment patterns in MS^2^ spectrum, we can discriminate complicate phenolic metabolites with similar structures in pecan organs clearly and efficiently.

**FIGURE 6:**
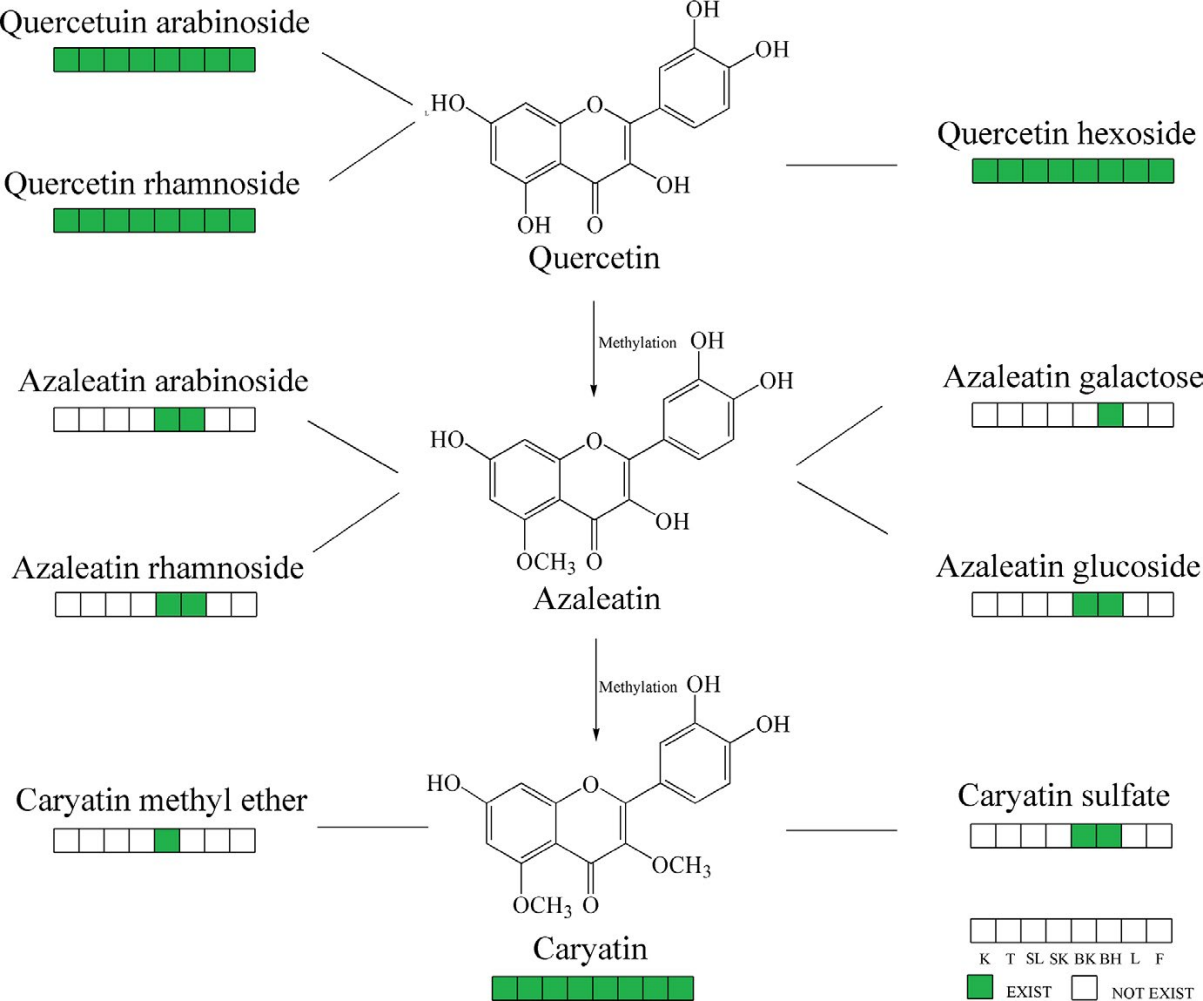
The distributions of flavonols in pecan organs. K, kernel; T, testa; SL, shell; SK, shuck; BK, bark; BH, branch; L, leaf; F, flower (male inflorescence)

Flavonoids reported in pecan previously were all isolated from pecan leaves, branches or barks, their structures were elucidated by NMR methods, so this is the first report of identifying them in other organs and this is also the first report of identifying them with LCMS method. Through comprehensive survey of pecan organs, some distribution patterns of flavonols in pecan were found (Figure 6). The flavonol aglycones quercetin, azaleatin, and caryatin found in pecan have similar structures, methylation of the 6‐hydroxy group of quercetin forms azaleatin, and further methylation of the 3‐hydroxy group of azaleatin forms caryatin. A lot of flavonols can only be found in bark, leaf, or branch, especially azaleatin derivatives and caryatin derivatives. These results might indicate that more methylations might occur in organs which had more chances to face biotic stresses like insects or diseases.

#### 3.2.4 Identification of other phenolic compounds and their distribution

The structure of peaks 28 and 41 ([M−H]^−^, 1,207) was much special, they were identified as complex tannins, which means a flavonol‐based motif linked with ellagitannins through C‐C bond (Okuda et al., 2009). By analysis of the fragmentation pattern, they were identified as stenophyllanin A/B isomer, which contained two HHDP, one galloyl and one epicatechin groups. This is the first report of this complex tannin in pecan. Brevifolin carboxylic acid (peak 39) and neochlorogenic acid (peak 16) were also assigned according to their high‐accuracy MWs, fragmentation patterns and compared with literature reports (Regueiro et al., 2014). Neochlorogenic acid was found only in pecan leaf in this experiment, and this is the first report of this compound in pecan.

### 3.3 Content

In order to get more accurate result, we use the UHPLC‐QQQ‐MS^n^ equipment with MRM mode to carry out the quantitative analysis (Table 2). The precursor and product ions of selected compounds for MRM quantification were showed in Table S3. Catechin and ellagic acid were quantified with standard compounds. Other phenolics were semi quantified with catechin and ellagic acid according to their structural characteristics, which means that ellagic acid derivatives using ellagic acid as reference, while condensed tannins and flavonols using catechin.

**TABLE 2:** Phenolic metabolite contents of pecan organs (mean ± *SD*, µg/g)

Peak No.	Phenolic metabolites	Kernel	Testa	Shell	Shuck	Bark	Branch	Leaf	Flower
Hydrolysable tannins									
Ellagitannins									
74	Ellagic acid	4.96 ± 0.04	29.75 ± 2.38	19.73 ± 3.96	2.04 ± 0.65	0.27 ± 0.06	6.57 ± 1.05	1.29 ± 0.03	3.56 ± 0.92
90	Methyl ellagic acid	27.00 ± 9.80	369.64 ± 53.53	76.28 ± 10.43	7.76 ± 0.66	42.56 ± 5.53	41.33 ± 36.06	21.33 ± 0.92	8.76 ± 2.66
59	Ellagic acid pentose	515.77 ± 167.20	2,882.85 ± 831.87	390.92 ± 36.55	59.61 ± 7.72	9.65 ± 1.98	356.53 ± 75.30	447.62 ± 6.76	39.99 ± 7.23
85	Ellagic acid rhamnoside	273.87 ± 129.00	1,727.29 ± 304.49	111.71 ± 42.21	6.31 ± 1.06	4.80 ± 0.36	2,590.89 ± 229.48	268.85 ± 9.23	40.64 ± 5.27
83	Methyl ellagic acid pentose	71.81 ± 20.42	349.10 ± 87.00	57.79 ± 7.69	9.06 ± 0.92	2.33 ± 0.08	256.25 ± 53.71	26.06 ± 5.24	42.9 ± 2.71
92	Dimethyl ellagic acid rhamnoside	52.02 ± 15.58	280.46 ± 26.15	224.80 ± 52.68	3.37 ± 0.34	0.58 ± 0.09	8.78 ± 3.18	12.85 ± 0.47	1.07 ± 0.07
76	Methyl ellagic acid glucose	2.45 ± 0.24	4.43 ± 1.23	44.93 ± 12.56	62.38 ± 29.83	14.15 ± 4.30	123.78 ± 15.60	363.90 ± 21.71	189.30 ± 24.31
3	HHDP‐glucose	76.08 ± 18.14	1,161.76 ± 322.21	53.49 ± 21.77	0.85 ± 0.23	0.15 ± 0.06	258.06 ± 78.58	297.41 ± 4.66	62.97 ± 8.25
2	HHDP‐glucose	5.87 ± 0.70	3.20 ± 0.75	0.18 ± 0.09	0.02 ± 0.04	0.01 ± 0.0003	1.19 ± 0.48	0.77 ± 0.04	0.19 ± 0.04
1	HHDP‐glucose	71.51 ± 24.84	957.46 ± 138.33	48.41 ± 22.52	2.25 ± 0.24	0.21 ± 0.07	126.45 ± 14.93	131.30 ± 9.18	24.50 ± 3.70
12	Pedunculagin/casuariin isomer	59.50 ± 35.37	828.07 ± 28.96	30.40 ± 16.31	2.45 ± 0.50	0.10 ± 0.02	100.60 ± 15.33	83.50 ± 0.73	95.80 ± 14.55
18	Pedunculagin/casuariin isomer	0.12 ± 0.03	1.08 ± 0.32	0.03 ± 0.001	0.01 ± 0.01	ND	0.18 ± 0.06	2.37 ± 0.09	0.08 ± 0.003
Hydrolyzed tannins mixed with both HHDP and galloyl groups									
97	Methyl ellagic acid galloyl pentose	4.40 ± 0.94	79.17 ± 8.67	3.90 ± 1.16	0.29 ± 0.05	0.05 ± 0.01	1.69 ± 0.80	1.60 ± 0.18	0.82 ± 0.36
95	Methyl ellagic acid galloyl pentose	44.46 ± 2.33	645.46 ± 88.63	54.62 ± 16.06	9.19 ± 0.83	0.56 ± 0.07	13.30 ± 3.74	69.65 ± 3.68	16.03 ± 2.55
96	Methyl ellagic acid galloyl pentose	4.38 ± 0.33	53.73 ± 9.26	6.07 ± 1.45	0.75 ± 0.12	0.06 ± 0.01	1.44 ± 0.65	6.27 ± 0.43	1.53 ± 0.39
8	Valoneoyl‐glucose	0.29 ± 0.14	2.93 ± 0.53	0.13 ± 0.09	0.01 ± 0.01	ND	0.35 ± 0.06	1.28 ± 0.17	0.10 ± 0.01
25	Tellimagrandin I	3.62 ± 1.55	48.99 ± 3.12	1.35 ± 0.35	0.05 ± 0.01	0.01 ± 0.01	0.95 ± 0.53	1.30 ± 0.09	0.67 ± 0.07
34	Casuarinin/casuarictin isomer	0.67 ± 0.30	30.39 ± 3.59	4.80 ± 0.47	0.44 ± 0.06	0.03 ± 0.01	0.88 ± 0.29	4.20 ± 0.22	3.41 ± 1.00
22	Praecoxin A/platycariin isomer	4.90 ± 0.90	80.06 ± 11.47	14.38 ± 1.98	2.47 ± 0.41	0.28 ± 0.03	1.03 ± 0.26	47.60 ± 3.03	5.66 ± 1.51
37	Praecoxin A/platycariin isomer	5.39 ± 0.91	78.72 ± 3.06	15.71 ± 1.09	2.76 ± 0.33	0.31 ± 0.02	3.68 ± 0.14	50.68 ± 6.27	5.21 ± 1.44
19	Praecoxin A/platycariin isomer	2.64 ± 1.37	41.66 ± 7.81	12.75 ± 0.57	2.37 ± 0.39	0.21 ± 0.03	1.13 ± 0.28	127.85 ± 3.77	8.84 ± 2.57
41	Stenophyllanin A/B isomer	29.7 ± 7.17	129.88 ± 27.79 *	9.87 ± 1.24	5.10 ± 1.60	0.49 ± 0.23	24.03 ± 3.17	9.10 ± 1.18	21.55 ± 7.59
Total content of hydrolysable tannins		1,261.41	9,786.08	1,086.24	169.74	76.81	3,871.19	1,949.78	561.26
Condensed tannins									
27	(+)‐Catechin	6.02 ± 0.65	35.61 ± 3.25	2.67 ± 1.28	29.99 ± 4.08	1.90 ± 0.34	4.10 ± 1.91	5.40 ± 0.66	0.80 ± 0.04
43	Procyanidin dimer B linkage	3.51 ± 0.47	50.50 ± 3.06	1.85 ± 0.65	56.63 ± 1.62	3.59 ± 0.45	2.43 ± 1.22	3.85 ± 0.51	0.84 ± 0.01
20	Procyanidin dimer B linkage	0.94 ± 0.12	13.58 ± 1.17	0.63 ± 0.22	5.86 ± 0.41	0.65 ± 0.15	0.27 ± 0.06	1.13 ± 0.06	0.03 ± 0.0007
24	Procyanidin dimer B linkage	0.01 ± 0.0006	0.12 ± 0.02	0.01 ± 0.01	0.02 ± 0.003	0.01 ± 0.002	ND	ND	0.01 ± 0.003
14	1 (E)C and 1 (E)GC B linkage	2.86 ± 0.25	90.90 ± 6.48	31.19 ± 10.00	5.78 ± 0.78	0.19 ± 0.06	0.24 ± 0.11	5.95 ± 0.54	0.11 ± 0.04
Total content of condensed tannins		13.34	190.71	36.35	98.28	6.34	7.04	16.33	1.79
Flavonols									
65	Azaleatin rhamnoside	0.54 ± 0.10	5.46 ± 0.35	0.18 ± 0.05	ND	ND	0.07 ± 0.003	0.52 ± 0.01	0.07 ± 0.002
87	Caryatin	2.30 ± 1.59	1.04 ± 1.00	16.19 ± 14.73	6.23 ± 0.83	665.17 ± 127.07	1,156.38 ± 378.92	0.33 ± 0.15	0.20 ± 0.05
75	Quercetin hexoside	0.07 ± 0.07	0.44 ± 0.11	0.11 ± 0.06	5.38 ± 1.59	0.17 ± 0.01	0.69 ± 0.11	57.33 ± 9.57	5.27 ± 0.50
86	Quercetin rhamnoside	0.38 ± 0.13	3.10 ± 0.40	0.48 ± 0.25	4.86 ± 1.18	2.46 ± 0.39	66.04 ± 4.90	250.46 ± 31.17	23.61 ± 3.37
Total content of flavonols		3.29	10.04	16.96	16.47	667.80	1,223.18	308.64	29.15
Total content of phenolics quantified with LCMS		1,278.04	9,986.83	1,139.55	284.49	750.95	5,101.41	2,274.75	592.20
								
Total phenolic content (mg EAE g^−1^)		11.39 ± 5.08	132.27 ± 24.87	44.00 ± 5.91	4.84 ± 0.90	23.72 ± 2.52	11.79 ± 3.30	26.00 ± 2.95	24.96 ± 5.74
DPPH (μmol TE g^−1^)		71.49 ± 20.43	9,435.93 ± 624.49	3,269.21 ± 863.34	109.63 ± 25.03	416.54 ± 179.51	243.38 ± 28.32	547.28 ± 127.39	496.82 ± 190.15
ABTS (μmol TE g^−1^)		182.85 ± 27.13	8,493.96 ± 1,860.77	3,439.00 ± 90.36 ±4.52	271.81 ± 16.48	1,020.05 ± 175.43	721.54 ± 64.70	1,407.17 ± 324.07	1,119.02 ± 125.70

Several phenolics' contents were really high in pecan, such as ellagic acid pentose in testa (2,882.85 µg/g), ellagic acid rhamnoside in branch (2,590.89 µg/g) and testa (1,727.29 µg/g), HHDP‐glucose in testa (1,161.76 µg/g), and caryatin in branch (1,156.38 µg/g).

The predominant phenolics in different pecan organs were different, such as the top five phenolics in pecan testa were ellagic acid pentose (2,882.85 µg/g), ellagic acid rhamnoside (1,727.29 µg/g), HHDP‐glucose (peak 3, 1,161.76 µg/g), HHDP‐glucose (peak 1, 957.46 µg/g) and pedunculagin/casuariin isomer (1,161.76 µg/g), while the top five in bark were caryatin (665.17 µg/g), methyl ellagic acid (42.56 µg/g), methyl ellagic acid glucose (14.15 µg/g), ellagic acid pentose (9.65 µg/g), and ellagic acid rhamnoside (4.80 µg/g), but ellagic acid pentose was one of the top five phenolics in all organs.

The total content of hydrolysable tannins was significantly high in pecan testa (9,786.08 µg/g), branch (3,871.19 µg/g), leaf (1,949.78 µg/g), and kernel (1,261.41 µg/g), but low in bark and shuck. The total content of condensed tannins was much lower than that of hydrolysable tannins, their content was varying from 190.71 µg/g to 1.79 µg/g, highest content appeared in testa, while lowest in flower. Combined with previous qualification results, we can see that tannins tend to accumulate in pecan testa with both diverse structures and high contents.

The distribution of flavonols was much different with tannins, they were the prevailing phenolics in branch (1,223.18 µg/g), bark (667.80 µg/g), and leaf (308.64 µg/g), but rare in testa (10.04 µg/g) and kernel (3.29 µg/g). This result consisted with previously reports while most flavonols were isolated and identified firstly from pecan branch, bark or leaves (Abdallah et al., 2011; Gad et al., 2007; Ishak et al., 1980). The highest content among flavonols appeared at 1,156.38 µg/g of caryatin in branch, followed by 665.17 µg/g of caryatin in bark and 250.46 µg/g of quercetin rhamnoside in leaf. Combined with previous results, we can see that flavonols tend to accumulate in organs such as branch, bark, or leaf in pecan with both diverse structures and high contents.

The total content of all phenolics quantified with LCMS was highest in testa, so were the TPC and antioxidant capacities (Table 2), all these results indicated that pecan testa contained high concentration of phenolics. Such high concentration of phenolics plays important roles in protecting pecan kernel from invasion of diseases and insects. The total content of phenolics quantified with LCMS was lowest in shuck, so was the TPC. The TPC and antioxidant capacities of pecan shell were also relatively high, which is consistent with previous results. At present, the research on antioxidant capacities of pecan is limited to kernel and shell (Biomhoff et al., 2006; de la Rosa et al., 2011, 2014; Prado et al., 2013, 2014; Flores‐Cordova et al., 2017; Hilbig, Alves, et al., 2018; Jia et al., 2018; Robbins et al., 2015; Villarreal‐Lozoya et al., 2007; Wu et al., 2004). Although there were some differences among different research results due to the differences of producing areas, varieties, and extraction methods, the antioxidant capacities of shell were higher than that of kernel in all reports, about 5–7 times of that of kernel. In this experiment, we separated the testa from kernel, which provided a more accurate result of different pecan organs.

## 4 CONCLUSIONS

The composition and distribution of phenolic metabolites in eight different pecan organs were analyzed for the first time. A rapid qualitative method of LTQ‐Orbitrap MS and an accurate quantitative method of QQQ MS were established. Ninety‐seven phenolics were identified from eight pecan organs. Twelve phenolics were identified for the first time in pecan, including dimethyl ellagic acid rhamnoside, pterocarinin B, glansrin C, casuarinin/casuarictin isomer, pterocarinin A, eucalbanin A/cornusiin B isomer, rugosin C/glansrin A isomer, stenophyllanin A/B isomer, tris‐O‐degalloyl rugosin F isomer, reginin A/D isomer, heterophylliin D, and neochlorogenic acid. With the help of high‐accuracy MW, phenolic metabolites with similar MW and structures in pecan organs can be discriminated more clearly and accurately. Because the previous researches were mainly focused on pecan kernels, so this is the first report of many phenolic metabolites in other organs. The contents of thirty‐three phenolics were determined under MRM mode. Combined with the previous qualification, the compositions of phenolic metabolites in different pecan organs were more clearly. A massive phenolic metabolites' matrix in different pecan organs was built in this experiment, which should be useful for related researches in the future and help provide a theoretical basis for using these organs as functional foods.

## CONFLICT OF INTEREST

The authors declare that they have no conflict of interests.

## ETHICAL APPROVAL

The human and animal testing was unnecessary in the current study.

## Supporting information

Table S1

Table S2

Table S3
