# Supplementary material for: Metabolic profiling revealed the organ‐specific distribution differences of tannins and flavonols in pecan
Source: Food Sci Nutr. 2020 Aug 10;8(9):4987–5006. doi: 10.1002/fsn3.1797 (PMC7500802; doi:10.1002/fsn3.1797)
Supplement: Supplementary file 1 — Table S1 [file FSN3-8-4987-s001.docx]

**Table S1.** Linearity, LOD and LOQ of standard compounds

| **Compounds** | **Regression equation** | **r^2^** | **Linear range (μg·mL^-1^)** | **LOD (μg·mL^-1^)** | **LOQ (μg·mL^-1^)** |
| --- | --- | --- | --- | --- | --- |
| Catechin | y = 0.0003x + 1.3756 | 0.9907 | 0.0077-151.0 | 0.0009 | 0.0029 |
| Ellagic acid | y = 0.0006x - 0.0526 | 0.9905 | 0.2285-117.0 | 0.0008 | 0.0026 |
| Quercetin | y = 6E-05x + 0.0636 | 0.9994 | 0.0052-103.0 | 0.0008 | 0.0028 |

* y, peak area; x, compound concentration (μg·mL^-1^); LOD=limit of detection, S/N=3; LOQ=limit of quantitation, S/N=10.
