# Supplementary material for: Metabolic profiling revealed the organ‐specific distribution differences of tannins and flavonols in pecan
Source: Food Sci Nutr. 2020 Aug 10;8(9):4987–5006. doi: 10.1002/fsn3.1797 (PMC7500802; doi:10.1002/fsn3.1797)
Supplement: Supplementary file 2 — Table S2 [file FSN3-8-4987-s002.docx]

**Table S2.** Precision, repeatability and stability of standard compounds

| **Compounds** | **Precision (RSD, %)** | **Repeatability** | **Stability**  **(RSD, %, n=6)** |
| --- | --- | --- | --- |
|  | **Intraday (n=6)** | **(RSD, %, n=6)** |  |
| Catechin | 0.6348 | 0.9675 | 1.9626 |
| Ellagic acid | 1.8024 | 2.5389 | 2.7974 |
| Quercetin | 1.3244 | 2.2881 | 2.2997 |
