# Supplementary material for: Metabolic profiling revealed the organ‐specific distribution differences of tannins and flavonols in pecan
Source: Food Sci Nutr. 2020 Aug 10;8(9):4987–5006. doi: 10.1002/fsn3.1797 (PMC7500802; doi:10.1002/fsn3.1797)
Supplement: Supplementary file 3 — Table S3 [file FSN3-8-4987-s003.docx]

**Table S3.** List of quantified compounds, MRM parameters (retention times, precursor and product ions *m/z*)

| **Peak No.** | **RT (min)** | **Precursor ion (Q1)** | **Product ion (Q3)** | **Formula** | **Compound identification** |
| --- | --- | --- | --- | --- | --- |
| **Hydrolysable tannins** | | | | | |
| Ellagitannins | | | | | |
| 74 | 8.22 | 300.9998 | 161 | C_14_H_6_O_8_ | Ellagic acid |
| 90 | 10.15 | 315.0148 | 300 | C_15_H_8_O_8_ | Methyl ellagic acid |
| 59 | 7.60 | 433.0428 | 301 | C_19_H_14_O_12_ | Ellagic acid pentose |
| 85 | 9.46 | 447.0579 | 315 | C_20_H_16_O_12_ | Ellagic acid rhamnoside |
| 83 | 9.28 | 447.0581 | 300 | C_20_H_16_O_12_ | Methyl ellagic acid pentose |
| 92 | 10.87 | 475.0888 | 329 | C_22_H_20_O_12_ | Dimethyl ellagic acid rhamnoside |
| 76 | 8.39 | 477.0690 | 301 | C_21_H_18_O_13_ | Methyl ellagic acid hexoside |
| 3 | 1.10 | 481.0628 | 301 | C_20_H_18_O_14_ | HHDP-glucose |
| 2 | 0.95 | 481.0632 | 191 | C_20_H_18_O_14_ | HHDP-glucose |
| 1 | 0.72 | 481.0641 | 301 | C_20_H_18_O_14_ | HHDP-glucose |
| 12 | 3.02 | 783.0690 | 301 | C_34_H_24_O_22_ | Pedunculagin/casuariin isomer |
| 18 | 4.25 | 783.0690 | 633 | C_34_H_24_O_22_ | Pedunculagin/casuariin isomer |
| Hydrolyzed tannins mixed with both HHDP and galloyl groups | | | | | |
| 97 | 12.55 | 599.0685 | 300 | C_27_H_20_O_16_ | Methyl ellagic acid galloyl pentose |
| 95 | 11.93 | 599.0687 | 447 | C_27_H_20_O_16_ | Methyl ellagic acid galloyl pentose |
| 96 | 12.17 | 599.0687 | 300 | C_27_H_20_O_16_ | Methyl ellagic acid galloyl pentose |
| 8 | 1.72 | 649.0694 | 605 | C_27_H_22_O_19_ | Valoneoyl-glucose |
| 25 | 5.12 | 785.0854 | 483 | C_34_H_26_O_22_ | Tellimagrandin I |
| 34 | 5.72 | 935.0845 | 301 | C_41_H_28_O_26_ | Casuarinin/casuarictin isomer |
| 22 | 4.85 | 951.0759 | 783 | C_41_H_28_O_27_ | Praecoxin A/platycariin isomer |
| 37 | 5.97 | 951.0782 | 783 | C_41_H_28_O_27_ | Praecoxin A/platycariin isomer |
| 19 | 4.53 | 951.0789 | 783 | C_41_H_28_O_27_ | Praecoxin A/platycariin isomer |
| 41 | 6.26 | 1207.1397 | 917 | C_56_H_40_O_31_ | Stenophyllanin A/B isomer |
| **Condensed tannins** | | | | | |
| 27 | 5.28 | 289.0726 | 161 | C_15_H_14_O_6_ | (+)-Catechin |
| 43 | 6.38 | 577.1357 | 407 | C_30_H_26_O_12_ | Procyanidin dimer B linkage |
| 20 | 4.73 | 577.1364 | 425 | C_30_H_26_O_12_ | Procyanidin dimer B linkage |
| 24 | 5.01 | 577.1372 | 301 | C_30_H_26_O_12_ | Procyanidin dimer B linkage |
| 14 | 3.69 | 593.1309 | 407 | C_30_H_26_O_13_ | 1 (E)C and 1 (E)GC B linkage |
| **Flavonols** | | | | | |
| 65 | 7.91 | 461.1106 | 315 | C_22_H_22_O_11_ | Azaleatin rhamnoside |
| 87 | 9.70 | 329.0671 | 271 | C_17_H_14_O_7_ | Caryatin |
| 75 | 8.31 | 463.0897 | 301 | C_21_H_20_O_12_ | Quercetin hexoside |
| 86 | 9.58 | 447.0949 | 301 | C_21_H_20_O_11_ | Quercetin rhamnoside |
